# Shared and distinct mechanisms of iron acquisition by bacterial and fungal pathogens of humans

**DOI:** 10.3389/fcimb.2013.00080

**Published:** 2013-11-19

**Authors:** Mélissa Caza, James W. Kronstad

**Affiliations:** The Michael Smith Laboratories, Department of Microbiology and Immunology, University of British ColumbiaVancouver, BC, Canada

**Keywords:** heme, hemoglobin, transferrin, siderophores, iron, microbial pathogenesis

## Abstract

Iron is the most abundant transition metal in the human body and its bioavailability is stringently controlled. In particular, iron is tightly bound to host proteins such as transferrin to maintain homeostasis, to limit potential damage caused by iron toxicity under physiological conditions and to restrict access by pathogens. Therefore, iron acquisition during infection of a human host is a challenge that must be surmounted by every successful pathogenic microorganism. Iron is essential for bacterial and fungal physiological processes such as DNA replication, transcription, metabolism, and energy generation via respiration. Hence, pathogenic bacteria and fungi have developed sophisticated strategies to gain access to iron from host sources. Indeed, siderophore production and transport, iron acquisition from heme and host iron-containing proteins such as hemoglobin and transferrin, and reduction of ferric to ferrous iron with subsequent transport are all strategies found in bacterial and fungal pathogens of humans. This review focuses on a comparison of these strategies between bacterial and fungal pathogens in the context of virulence and the iron limitation that occurs in the human body as a mechanism of innate nutritional defense.

## Introduction

Iron is an extremely versatile cofactor that is essential for many biochemical reactions in both mammalian hosts and pathogenic microbes. Ferrous (Fe^2+^) and ferric (Fe3^+^) iron, the biologically relevant forms, are found in prosthetic groups, such as iron-sulfur clusters and heme, that are incorporated into many metalloproteins (e.g., aconitase and cytochromes), where the iron serves as a biocatalyst or as an electron carrier. Iron is also found in many mono- and di-nuclear non–heme iron proteins like ferritin and ribonucleotide reductase. The redox potential of iron makes it especially useful for biological processes, in particular for oxidative phosphorylation where iron reduction/oxidation facilitates electron transfer in the respiratory chain. Moreover, iron is present in multiple proteins with diverse functions that include replication and repair of DNA, transport of oxygen, metabolism of carbon [e.g., via the tricarboxylic acid (TCA) cycle] and regulation of gene expression. Several reviews on the importance of iron in biological processes have appeared recently (Evstatiev and Gasche, 2012; Tandara and Salamunic, 2012; Dlouhy and Outten, 2013; Ilbert and Bonnefoy, 2013).

Because of its utility, iron is an essential element and an object of extreme competition between pathogens and their hosts. However, upon oxygenation of the Earth's atmosphere, the predominant form of iron switched from the relatively soluble ferrous state to the extremely insoluble ferric form at neutral pH. In fact, ferric iron is oxidized and polymerized into insoluble polymers of ferric (oxy)hydroxide at pH 7.0, thus further limiting its biological accessibility (Griffiths, 1999; Ilbert and Bonnefoy, 2013). On the other hand, ferrous iron is quite toxic due to its propensity to react with oxygen to generate reactive oxygen species (ROS) via the Fenton and Haber-Weiss reactions. ROS can damage membrane lipids, proteins and DNA (Imlay, 2003). Therefore, iron acquisition, storage, and incorporation into proteins must be carefully managed by mechanisms that promote solubility, control the redox state, and avoid toxicity.

In this review, we discuss and compare selected examples of how pathogenic bacteria and fungi perform iron uptake in the context of competitive sequestration by host proteins. Detailed studies have been performed in a large number of bacterial species and we will focus on illustrative examples. For the fungi, we will describe iron acquisition systems in the three best-studied opportunistic pathogens. These are the mold *Aspergillus fumigatus* (a saprotroph that is also responsible for invasive pulmonary aspergillosis), the polymorphic fungus *Candida albicans* (the cause of skin or mucosal infections and invasive candidiasis), and the yeast *Cryptococcus neoformans* (the agent of cryptococcosis, a disease involving life-threatening meningoencephalitis). We have mainly focused our discussion on iron sources and uptake mechanisms in the context of virulence, with limited coverage of regulation. This is because many excellent reviews have summarized regulatory aspects of iron acquisition and homeostasis in bacteria, fungi and mammals (Andrews et al., 2003; Hentze et al., 2010; Cornelis et al., 2011; Schrettl and Haas, 2011; Wang and Pantopoulos, 2011; Pantopoulos et al., 2012; Philpott et al., 2012; Salvail and Masse, 2012; Kronstad et al., 2013).

## Iron distribution in the mammalian host: opportunities for microbial exploitation

A large quantity of iron is potentially available to microbes upon infection of vertebrate hosts, although pathogens must extract this iron from a variety of proteins in blood, different cell types, and tissue locations. In fact, the average human adult contains 3–5 g of iron, the majority of which (65–75%) is found in heme associated with hemoglobin within erythrocytes (red blood cells or RBCs) (McCance and Widdowson, 1937; Andrews, 2000). Each day, 20–25 mg of iron is required to support the synthesis of hemoglobin. The daily intake of iron is very low (1–2 mg per day); therefore a considerable amount of iron is recycled each day mainly by macrophages. Macrophages recognize and phagocytose damaged or senescent RBCs, with the spleen playing a major role in recycling. Phagocytized RBCs are first degraded to extract heme and iron is subsequently released by heme oxygenase (HO-1) for reutilization in erythropoiesis. Hence, approximately 1 g of iron is stored in hepatocytes and macrophages of the liver (Kupffer cells) and spleen. A number of recent reviews have appeared that summarize iron homeostasis in humans (Bleackley et al., 2009; Evstatiev and Gasche, 2012; Ganz, 2012; Tandara and Salamunic, 2012).

Dietary iron is taken up in the intestine (duodenum and upper jejunum) either as ferrous iron [after reduction of ferric iron by the intestinal ferric reductase, duodenal cytochrome B (DcytB)], or as heme (McKie et al., 2001; Latunde-Dada et al., 2008; Evstatiev and Gasche, 2012). The ferrous iron is transported by divalent metal transporter 1 (DMT1), located at the apical membrane of enterocytes (Fleming et al., 1997; Gunshin et al., 1997). The mechanism of dietary heme uptake remains to be clarified. The heme carrier protein 1 (HCP1) was proposed as a heme receptor in duodenal enterocytes (Shayeghi et al., 2005); however, its primary role may be to transport folic acid rather than heme (Qiu et al., 2006). HRG-1, the heme responsive gene-1, was first identified in *Caenorhabditis elegans* as a heme importer (Rajagopal et al., 2008). The human homologue of HRG-1 appears to transport heme as well, but rather from the lysosome into the cytosol (Yanatori et al., 2010; Delaby et al., 2012). FLVCR2 (feline leukemia virus, subgroup C receptor 2) was also recently reported to mediate the endocytosis of heme by mammalian cells (Duffy et al., 2010). The availability of dietary iron to pathogens and the microbiota in the intestine is relevant to colonization, commensalism, and invasion, as demonstrated by recent studies with both bacterial and fungal pathogens (Chen et al., 2011; Kortman et al., 2012; Deriu et al., 2013).

Iron can also be found in blood upon the release of hemoglobin and heme from ruptured erythrocytes and enucleated erythroblasts. However, free hemoglobin is trapped by haptoglobin and taken up by hepatocytes or macrophages via the CD163 receptor (Kristiansen et al., 2001). Heme that is released into the bloodstream can also be bound by hemopexin, albumin, and high and low density lipoproteins (HDL and LDL) (Ascenzi et al., 2005). The hemopexin-heme complex is cleared by hepatocytes and macrophages via the CD91 receptor (Hvidberg et al., 2005). Plasma heme can also originate from the degradation of myoglobin and heme-containing enzymes such as catalases, peroxidases and cytochromes, and from myeloperoxidase secreted from neutrophils (Ascenzi et al., 2005). All these mechanisms promote iron recycling and also protect the host from iron toxicity.

Transferrin in the circulatory system can also potentially be exploited by microbes during bloodstream infections. Approximately 2–3 mg of iron is bound to partly saturated transferrin in plasma (Tandara and Salamunic, 2012). However, transferrin in serum is partially saturated (about 30–40%) to limit the availability of free iron (Williams and Moreton, 1980; Aisen et al., 2001). The transferrin polypeptide has two homologous globular lobes that each binds one iron atom, and ferric iron is tightly bound at physiological pH (*K*_*a*_ about 10^20^ M^−1^) (Aisen and Brown, 1977). Consequently, the plasma concentration for free ferric iron is ~10^−24^ M (Otto et al., 1992). Transferrin delivers ferric iron to cells via the transferrin receptor (TfR1) expressed on almost every cell, and also by another receptor, TfR2, expressed in hepatocytes (Hu and Aisen, 1978; Kawabata et al., 1999; Fleming et al., 2000). Iron-loaded transferrin bound to its receptor is endocytosed through a clathrin-dependent pathway, and acidification during endosome maturation dissociates ferric iron from transferrin; the iron-depleted complex is then recycled (Dautry-Varsat et al., 1983; Aisen, 2004; Steere et al., 2012). Subsequent reduction of iron to the ferrous form is achieved in endosomes by the Steap 3 (six-transmembrane epithelial antigen of the prostate 3) protein in erythrocytes and other Steap proteins in non-erythroid cells (Ohgami et al., 2005, 2006). Iron is exported from endosomes to the cytosol by DMT1 (Fleming et al., 1998).

Lactoferrin is a member of the transferrin family that is predominantly found in milk, but can also be present in mucosal secretions like tears and saliva, and in neutrophil granules (Evans et al., 1999). Like transferrin, lactoferrin can bind two atoms of iron, but it retains iron at a much lower pH (~3.0) than transferrin (~5.5) (Mazurier and Spik, 1980; Baker and Baker, 2004). Lactoferrin contributes to immunity by iron sequestration at sites of infection. Similarly, the host protein siderocalin (also called NGAL and lipocalin2) plays a role in the innate immune response against microbial pathogens by iron sequestration (Flo et al., 2004). It has been proposed that the small molecule 2,5-dihydroxybenzoic acid, also known as gentisic acid, functions as a mammalian siderophore (a low molecular weight iron chelator) (Devireddy et al., 2010). This molecule is able to bind iron and it was proposed that it delivers the metal to cells via interaction with the siderocalin and the cell surface receptor 24p3R (Devireddy et al., 2005). However, binding studies contradict this hypothesis, since gentisic acid does not form high-affinity complexes with siderocalin and iron (Correnti et al., 2012). The interesting role of siderocalin and it physiological importance in mammalian iron homeostasis are yet to be defined; however, its function in the competition for iron with bacterial pathogens is better understood and described in more detail below in the discussion of siderophores.

Once iron is taken into a cell, it is stored in ferritins for later use or incorporated into metalloproteins in complexes with heme (e.g., catalase, cytochromes, hemoglobin and myoglobin), as mono and dinuclear iron (e.g., ribonucleotide reductase), or as Fe-S clusters (e.g., aconitase, succinate dehydrogenase) (Rouault and Tong, 2008; Dlouhy and Outten, 2013). Ferritins are iron-storage proteins composed of 24 subunits and are able to accumulate up to 4500 iron atoms (Fischbach and Anderegg, 1965; Hoare et al., 1975). These proteins are present in the cytoplasm, nucleus, and mitochondria of cells and also in plasma, and they release iron during iron deficiency via a mechanism involving lysosome acidification and autophagy (De Domenico et al., 2006; Asano et al., 2011). Iron can be exported from cells by ferroportin, a ferrous iron transporter (Donovan et al., 2000). Ferrous iron can be oxidized by hephaestin in intestinal enterocytes and by ceruloplasmin in macrophages, immune cells and other cell types, and loaded onto transferrin for subsequent distribution via the bloodstream (Curzon and O'reilly, 1960; Osaki et al., 1966; Yang et al., 1986; Vulpe et al., 1999). Iron homeostasis in humans is maintained by the major regulator hepcidin that binds to ferroportin and promotes its degradation. This triggers a series of event resulting in a loss of intestinal iron absorption and cellular iron efflux (Anderson et al., 2002; Nemeth et al., 2004). The regulation of iron homeostasis has been reviewed recently (Evstatiev and Gasche, 2012; Tandara and Salamunic, 2012; Finberg, 2013).

**Figure 1:**
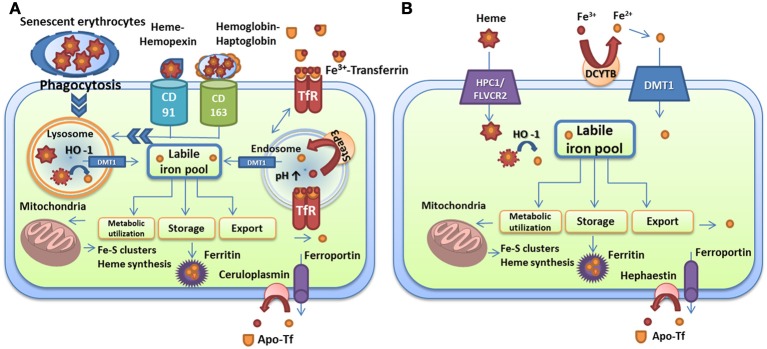
**Iron transport and homeostasis in human cells. (A)** Iron recycling in macrophages via phagocytosis of senescent red blood cells, uptake of heme-hemopexin and hemoglobin-haptoglobin complexes, and iron-loaded transferrin. **(B)** Dietary iron and heme absorption by intestinal endocytes via DMT1 and the heme receptor HCP1/FLVCR2, respectively. Iron-loaded siderocalin can also be absorbed via the receptor 24p3R. Iron is extracted from these carriers by heme oxygenase in lysosomes or by reductases in endosomes and is used for metabolic processes (mitochondria, storage, or export). Export is performed by ferroportin in partnership with ceruloplasmin in macrophages and with hephaestin in intestinal cells. Iron is loaded on transferrin for distribution. The descriptions of the specific proteins are given in the text.

The trafficking of iron in mammalian host cells is summarized in Figure 1. This figure and the information outlined above define the range of target iron sources that microbes can potentially exploit to proliferate in a variety of host tissues. It is clear that iron homeostasis and availability are tightly controlled by binding proteins and that the competition for iron is therefore a key aspect of infectious diseases. The microbial strategies to compete for iron are outlined in the following sections.

## Microbial strategies for iron acquisition from mammalian sources

Pioneering work by Schade and Caroline in 1944 revealed that high affinity iron binding proteins present in blood and egg whites are able to inhibit the growth of several bacterial species including *Escherichia coli*, as well as the yeast *Saccharomyces cerevisiae* (Schade and Caroline, 1944). They deduced that iron was too tightly bound to these proteins to be available to bacteria and yeast cells, thus inhibiting their growth. Importantly, growth could be restored by addition of iron, and this study was the first to establish a link between iron-related natural host resistance and microbial growth. Subsequently, Bullen et al. demonstrated that iron injection into guinea pigs considerably decreased the lethal dose of *E. coli*, thus suggesting an important role of iron in bacterial infection (Bullen et al., 1968). These and other studies led Kochan to propose the concept of “nutritional immunity,” the phenomenon that host control of access to essential nutrients, including iron, could impact the survival and proliferation of microbial pathogens (Kochan, 1973). In response, successful pathogens can overcome nutritional immunity by efficiently acquiring iron within the host via four strategies that target specific iron sources: (1) iron acquisition from heme and heme-containing proteins; (2) iron acquisition from transferrin, lactoferrin, and ferritin; (3) ferric iron acquisition by siderophores and; (4) uptake of ferrous iron. These strategies are described in the following sections.

## Iron acquisition from heme and heme-containing proteins

**Figure 2:**
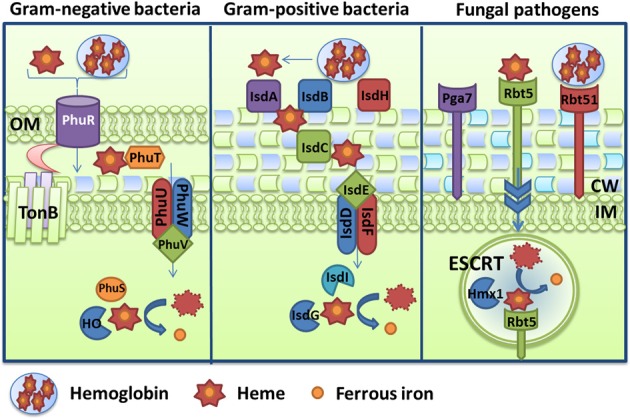
**Diagrams of hemoglobin and heme uptake and utilization.** Mechanisms are depicted for the Gram-negative bacterium *Pseudomonas aeruginosa* via the Phu system, and for the Gram-positive bacterium *Staphylococcus aureus* via the Isd system. For comparison, the scheme in the fungal pathogen *Candida albicans* is also illustrated and components include the receptors Rbt5, Rbt51, and Pga7. A schematic is also included to depict endocytosis (via ESCRT functions) and processing (with the heme oxygenase Hmx1). Additional details about the specific proteins are given in the text.

One strategy for microbes to obtain iron during infection of mammals is to target heme, hemoglobin, or complexes containing these molecules (e.g., haptoglobin-hemoglobin, hemopexin-heme). This strategy requires access to host heme sources, and several pathogenic bacteria and fungi therefore secrete hemolysins to lyse red blood cells and release hemoglobin, and/or produce hemoglobin proteases to degrade the protein. Hemolysins have been characterized in Gram-negative bacteria, such as pathogenic *E. coli* (α-hemolysin HlyA, ClyA, Hpb, and EspC) (Felmlee et al., 1985; Otto et al., 1998; Ludwig et al., 2004; Drago-Serrano et al., 2006), *Vibrio cholerae* El-Tor (HlyA) (Stoebner and Payne, 1988) and *Bordetella pertussis* (CyaA) (Glaser et al., 1988), as well as in Gram-positive bacteria including *Staphylococcus epidermis* (δ-hemolysin Hdl) (Verdon et al., 2009) and *Bacillus cereus* (hemolysin BL) (Senesi and Ghelardi, 2010). Fungi also produce hemolysins or have been reported to possess hemolytic activity. For example, the mold *A. fumigatus* secretes the hemolysin Asp which has hemolytic activity on chicken erythrocytes (Yokota et al., 1977). The polymorphic fungus *C. albicans* also possesses hemolytic activity but the yeast *C. neoformans* reportedly does not (Manns et al., 1994). Moreover, microbial pathogens have evolved two mechanisms to acquired iron from heme and heme-containing proteins: 1) direct uptake of heme and 2) use of hemophores (heme-binding proteins). These strategies have been extensively studied in numerous Gram-negative bacteria, while only a few examples are known in Gram-positive bacteria and in fungi. Iron acquisition strategies from heme and heme-containing proteins are illustrated in Figure 2 and described below.

### Heme uptake in bacteria

#### Gram-negative bacteria

The direct uptake of heme by Gram-negative bacteria is a well-characterized strategy for iron acquisition. In general, heme uptake is achieved by recognition and binding to a specific receptor in the outer membrane (OM). These surface receptors can directly bind heme and process its transport, but they are usually also able to bind heme-containing proteins like hemoglobin, haptoglobin-hemoglobin, hemopexin-heme, and myoglobin (Wandersman and Delepelaire, 2004). In that case, heme is extracted from these complexes and transported into the periplasm in a TonB-dependent manner (in Gram-negative bacteria). TonB-ExbB-ExbD is an energy-transducing complex that energizes outer membrane receptors to facilitate translocation of specific cargo (Braun and Braun, 2002). The majority of the outer membrane heme uptake receptors, as well as siderophore transporters and some transferrin/lactoferrin receptors (see below), are members of the TonB-dependent outer transporter (TBDT) family. Two unique domains are found in the TBDT fold: (1) a β-barrel with 22 anti-parallel strands and an internal diameter of 35–47 Å and (2) an N-terminal cork domain that blocks the internal space and prevents passive diffusion through the barrel (Ferguson and Deisenhofer, 2002). Furthermore, two His residues, or in some cases one Tyr residue, are required for heme transport across the barrel. Two conserved amino acid motifs (FRAP and NPNL) have also been identified on the extracellular loop containing one of the His residues (Bracken et al., 1999; Hagan and Mobley, 2009). The NPNL motif plays a role in binding heme due to its surface exposed location, whereas the FRAP motif is likely involved in heme transport across the cell membrane because it is buried inside the barrel (Liu et al., 2006). Once in the periplasm, heme is bound to a heme transport protein (HTP) and delivered to an ABC transporter in the inner membrane. Heme is then transported into the cytoplasm in an ATP-dependent fashion with subsequent degradation and iron release by bacterial heme oxygenases (Anzaldi and Skaar, 2010). The expression of the majority of these systems is controlled by the bacterial “ferric uptake regulator” protein Fur. Fur is a dimeric DNA-binding repressor that uses ferrous iron as a co-factor. Fur plays a central role in the bacterial response to iron starvation as it binds to promoter regions of iron-regulated genes at a so-called “fur box” and represses their expression under iron-replete conditions. Upon iron limitation, the Fur-Fe(II) complex dissociates from the DNA, thereby allowing transcription of iron-regulated genes (Hantke, 1981; Bagg and Neilands, 1987; De Lorenzo et al., 1987). Other proteins can also participate in the regulation of these systems under different conditions.

One of the two heme uptake system in *Pseudomonas aeruginosa* is illustrated in Figure 2 as an example. This system is encoded by the *phuR-phuSTUVW* genes, and PhuR is the outer membrane receptor, PhuT is the HTP, PhuUVW is the inner membrane ABC transporter and PhuS is an intracellular heme trafficking protein that delivers heme to the heme oxygenase (*pa*-HO, PigA, or HemO) (Ochsner et al., 2000; Ratliff et al., 2001; Lansky et al., 2006). This system facilitates the uptake of heme and the use of heme from hemoglobin since mutation of any component reduces growth on these iron sources (Ochsner et al., 2000). However, the specific mechanism of heme extraction from hemoglobin at the cell surface by PhuR or another protein is currently unknown. The amino acid sequence of PhuR shares similarity with several heme and hemoglobin receptors, such as HutA from *V. cholerae* (Henderson and Payne, 1994), ChuA *from E. coli* O157:H7 (Torres and Payne, 1997) and HmuR from *Y. pestis* (Hornung et al., 1996). Furthermore, three conserved motifs were identified in the amino acid sequence of PhuR, including a “TonB box,” and this strongly suggests a TonB-dependent translocation mechanism (Ochsner et al., 2000). Once heme is translocated into the periplasmic space, it is bound by PhuT. This heme transport protein binds heme and protoporphyrin IX at a ratio of 1:1 with high affinity (K_d_ ~ 1.2 and 14nM, respectively) (Tong and Guo, 2007). It is believe that PhuT delivers heme to the inner membrane transporter PhuUVW, although direct transfer of heme has not been demonstrated. Once in the cytoplasm, heme is bound by PhuS and delivered to the heme oxygenase HemO (Lansky et al., 2006). Protein-protein interaction studies identified a mechanism in which a heme-dependent conformational switch in PhuS drives heme release to HemO in a unidirectional fashion (Bhakta and Wilks, 2006; O'Neill et al., 2012). HemO is a δ-regioselective heme oxygenase that cleaves heme and produces biliverdin IX-β and -δ (Ratliff et al., 2001). Interestingly, the metabolic flux of heme uptake is driven by HemO, since mutation of the heme oxygenase results in loss of heme uptake and no production of biliverdin (Barker et al., 2012; O'Neill and Wilks, 2013). Expression of *phuR* and the *phuSTUVW* operon is controlled by the Fur regulator and two “Fur boxes” were identified by DNase footprinting (Ochsner et al., 2000).

Other similar heme and hemoglobin uptake systems have been characterized in several pathogenic Gram-negative bacteria including *Yersinia pestis* (HmuRSTUV) (Hornung et al., 1996; Thompson et al., 1999), *Yersinia enterocolitica* (HemRSTUV) (Stojiljkovic and Hantke, 1992, 1994), *Vibrio cholerae* (HutABCD) (Occhino et al., 1998) and the uropathogenic *E. coli* strain CFT073 (ChuA-Hma-DppABCDF) (Torres and Payne, 1997; Torres et al., 2001; Letoffe et al., 2006; Hagan and Mobley, 2009). Expression of the outer membrane receptors of these systems is regulated by Fur, they are all members of the TBDT family and they possess conserved FRAP and NPNL motifs. Site-direct mutagenesis of the TBDT HemR from *Y. enterocolitica* identified two conserved His residues as being required for heme transport through the receptor pore, while binding activity of heme was not affected (Bracken et al., 1999). The Hma receptor of *E. coli* requires a cell-surface exposed Tyr residue for heme use rather than the conserved His residues (Hagan and Mobley, 2009). The contributions of these systems to virulence have been evaluated for some of these bacteria. For example, virulence was tested for mutants lacking the Hmu and ChuA-Hma systems of *Y. pestis* and *E. coli*, respectively. Heme acquisition via the receptors ChuA and Hma in uropathogenic *E. coli* contributes to disease in mice, while the Hmu system in *Y. pestis* does not (e.g., when inoculated by subcutaneous or retro-orbital injection), presumably due to redundancy in iron acquisition systems for this species (Thompson et al., 1999; Torres et al., 2001; Hagan and Mobley, 2009).

Other systems are present in gram-negative bacteria for the use of hemoglobin as a sole source of iron. For example, *Haemophilus influenzae* type B (Hib) is able to use hemoglobin via three TonB-dependent cell surface receptors, HgpA, HgpB, and HgpC, that bind hemogloblin and hemoglobin-haptoglobin (Jin et al., 1996; Morton et al., 1999). Deletion of the *hgp* genes abolishes growth on hemoglobin-haptoglobin as a sole heme/iron source, although only a partial reduction occurred in the ability to use hemoglobin (Morton et al., 1999). The heme utilization protein Hup is responsible for this residual heme uptake activity from hemoglobin, since mutation of all of the *hgp* and *hup* genes resulted in a severe growth defect in the presence of low concentrations of hemoglobin or heme as the only iron source (Morton et al., 2004). It is believe that heme is extracted from hemoglobin at the cell surface by these receptors, although this activity has not yet been demonstrated. Nevertheless, once heme is translocated into the periplasm, it is taken up by the lipoprotein HbpA (Hanson and Hansen, 1991; Hanson et al., 1992b). Deletion of the *hpbA* gene in Hib resulted in growth diminution in the presence of low concentrations of heme, heme-hemopexin, and heme-albumin, but not in the presence of hemoglobin or hemoglobin-haptoglobin. These data indicate that *H. influenzae* may possess other periplasmic heme transporters in addition to HbpA (Morton et al., 2007b, 2009a). It has been proposed that HbpA delivers heme to the DppBCDF membrane transporter (Morton et al., 2009b), although several homologues of heme ABC transporters (SapACBDF and OppABCDF) have been discovered and may participate in heme transport in different strains of *H. influenzae* (Fleischmann et al., 1995; Mason et al., 2011). Nothing is known about how iron is extracted from heme once it enters the cytoplasm. Mutations in the *hgp*, *hup, hpbA* and *hel* (encoding lipoprotein *e* (P4), another periplasmic heme binding protein) genes had no impact on virulence in a bacteremia model with 5-day old rats (Morton et al., 2004, 2007a). However, mutation of the *hgp*, *hbpA*, and *hel* genes in Hib caused a significantly lower rate of bacteremia relative to the wild-type strain in a 30-day old rat model of infection (Seale et al., 2006; Morton et al., 2007b, 2009a). The level of plasma hemopexin and haptoglobin increases with age in rats, which may explain the requirement of different heme and hemoprotein acquisition systems for the virulence of *H. influenza* in older rats (Seale et al., 2006). This system of heme acquisition from hemoglobin is similar to the heme acquisition system from *P. aeruginosa* in that heme is transported into the cytoplasm by specific TonB-dependent outer membrane receptors, periplasmic proteins, and inner membrane ABC transporters.

Outer membrane receptors have also been identified that facilitate the use of hemoglobin. For example, *Neisseria meningitidis* is able to bind hemoglobin but not heme through the outer membrane receptor HmbR. This protein, like many outer membrane receptors for heme and iron acquisition systems, requires a functional TonB system and is regulated by Fur. HmbR functions by binding to hemoglobin and removing heme for subsequent translocation into the periplasm, and an NPNL motif has a possible role in heme removal. The cork domain of HmbR is also involved in heme passage to the periplasm (Perkins-Balding et al., 2003). Furthermore, an *hmbR* mutant is attenuated in an infant rat model for meningococcal infection, indicating that the use of hemoglobin as an iron source is important for *N. meningitidis* virulence (Stojiljkovic et al., 1995). *N. meningitidis* and *Neisseria gonorrhoeae* also possess a distinct bipartite TonB-dependent receptor for hemoglobin designated HpuAB. HpuB is an outer membrane receptor and HpuA encodes a lipoprotein, and together they transport heme from hemoglobin and the hemoglobin-haptoglobin complex. Expression of the *hpuAB* operon is regulated by iron and Fur (Lewis and Dyer, 1995; Lewis et al., 1997; Turner et al., 1998; Rohde et al., 2002). Both receptors (HmbR and HpuAB) are also subject to phase variation (Lewis et al., 1999) and the presence of either HmbR or HpuAB was found to be highly correlated with clinical isolates causing disease, suggesting a role in virulence for iron acquisition from hemoglobin (Tauseef et al., 2011). It is believe that these bacteria employ phase variation to more effectively adapt to the hostile environment of the host. So far, nothing is known about the intracellular transport of heme into the cytoplasm of these bacteria, although the process likely involves an ABC transporter. Similarly to the heme uptake system of *P. aeruginosa*, a heme oxygenase, HemO has been identified in *Neisseria* species and is required for the degradation of heme into ferric iron, biliverdin, and CO (Zhu et al., 2000a,b).

#### Gram-positive bacteria

Heme acquisition systems in Gram-positive bacteria share properties with those in Gram-negative bacteria in that they consist of cell surface receptors for heme, cell wall chaperone proteins that facilitate internalization of heme, ABC transporters that perform membrane translocation and heme oxygenase activities to release iron from heme. The HtaAB-HmuOTUV heme acquisition system identified in *Corynebacterium diphtheriae* illustrates the organization of one such system. Cell surface exposed HtaA binds hemoglobin and transfers heme to HtaB (Allen and Schmitt, 2009). Heme is believed to be transported inside the cell by the activities of the cell wall protein HmuT, the ATP transporter HmuUV and the cytoplasmic heme oxygenase HmuO that extracts the iron (Wilks and Schmitt, 1998; Drazek et al., 2000; Allen and Schmitt, 2009, 2011). Recently, another heme/hemoglobin system was identified in *C. diphtheriae* (Allen et al., 2013). Specifically, three proteins that are exposed on the cell surface, ChtA, ChtB, and ChtC, are able to bind heme and hemoglobin, with ChtA showing the highest affinity. A mutant lacking both *chtB* and *htaB* had significantly impaired iron use from heme, indicating a contribution of both systems for heme iron acquisition. No evaluations of virulence have been reported for these systems. As in Gram-negative bacteria, it appears that multiple heme acquisition systems are generally present in the Gram-positive bacteria characterized to date (i.e., with several surface receptors and ABC transporters).

### Use of hemophores by bacteria

#### Gram-negative bacteria

Hemophores are secreted proteins with the ability to bind heme and/or heme-containing proteins in the extracellular environment. This definition has recently been expanded to include any surface-exposed (or secreted) protein involved in the transfer of heme to a transporter for import (Wandersman and Delepelaire, 2012). A hemophore system was first discovered in 1994 in *Serratia marcescens* and others have been identified subsequently in Gram-negative and Gram-positive bacteria (Letoffe et al., 1994a; Wandersman and Delepelaire, 2012). As described below, a candidate hemophore has also recently been described in the fungal pathogen *C. neoformans* (Letoffe et al., 1994a; Cadieux et al., 2013). The hemophore system in *S. marcescens* (Has) includes the secreted HasA protein that is able to extract heme from hemoglobin, hemopexin and myoglobin (Letoffe et al., 1994a; Wandersman and Delepelaire, 2012). HasA is secreted by the export complex HasDEF, where HasD is an ATPase, HasE is a membrane fusion protein and HasF is an outer membrane protein (Letoffe et al., 1994b). Heme is transferred from hemoproteins to HasA by a passive mechanism due to higher affinity of HasA for heme, without protein-protein complex formation (Letoffe et al., 1999). HasA interacts with and delivers heme to the specific outer membrane receptor HasR (Izadi-Pruneyre et al., 2006). HasR can perform the uptake of heme from hemoglobin alone, but the process is 100 times more efficient with the participation of HasA (Arnoux et al., 2000). The determination of the structure of the HasR receptor revealed a cork and a β-barrel organization like other heme receptors, with two conserved His residues being important for heme binding (Izadi-Pruneyre et al., 2006; Krieg et al., 2009). This receptor actively transports heme with the help of HasB, a TonB orthologue that functions specifically with HasR (Benevides-Matos et al., 2008). After heme transfer from HasA to HasR, apo-HasA remains bound to HasR. The release of apo-HasA from the receptor is performed in an energy-driven process by HasB (Paquelin et al., 2001). This recycling process for HasA is only observed in the presence of heme, which is also required for the induction of *hasB* expression (Rossi et al., 2003; Wandersman and Delepelaire, 2012). The Has system is negatively regulated by iron and Fur, and positively regulated by a sigma and anti-sigma (HasI and HasS) signaling cascade triggered by heme-loaded hemophore binding to HasR (Rossi et al., 2003; Cwerman et al., 2006). Systems with similarity to Has have been reported in *P. aeruginosa* (Letoffe et al., 1998), *P. fluorescens* (Idei et al., 1999), and *Y. pestis* (Rossi et al., 2001). The contribution of the Has system to the virulence of *Y. pestis* has been assessed in a mouse model of bubonic plague and no role was found, even in the absence of the Hmu system for heme uptake (Rossi et al., 2001).

*H. influenza* type b (Hib) also produces a hemophore system (Hxu) that is synthesized from the *hxuCBA* gene cluster. The hemophore HxuA is able to bind the human heme-hemopexin complex and to release heme into the medium. HxuA is either anchored to the cell surface or partially released into culture medium depending on the strain (Wong et al., 1995). Unlike HasA, HxuA does not directly bind heme, but rather it interacts with hemopexin and interferes with its ability to sequester heme (Hanson et al., 1992a; Fournier et al., 2011). Free heme is then internalized by the TonB-dependent outer membrane receptor HxuC, while HxuB is involved in secretion of HxuA (Cope et al., 1995). It was also reported that HxuC is involved in residual use of heme from hemoglobin, as seen in an *hgp* triple knockout mutant, and in the direct use of heme from heme-albumin complexes. (Cope et al., 2001; Morton et al., 2007a). Moreover, deletion of the *hxuABC* genes significantly impaired the virulence of the strain in a 5-day-old rat model of bacteremia, but not in a 30-day old rat model, suggesting that these age related differences may be related to changes in levels of host heme-binding proteins during the development of the rat (Morton et al., 2007a). Subsequent heme transport across the inner membrane is likely to be performed by various ABC transporters as previously discussed.

Similar to HpuAB from *Neisseria* species, a bipartite receptor for heme has been described for *Porphyromonas gingivalis*. In this bacterium, the TonB-dependent heme receptor HmuR mediates heme uptake with the help of a heme-binding lipoprotein HmuY (Simpson et al., 2000; Olczak et al., 2008; Wojtowicz et al., 2009). HmuY has low affinity for heme but the proteolytic activity of secreted proteases (gingipains) on host heme-containing proteins facilitates heme release. For example, it has been demonstrated that HmuY can extract heme from hemoglobin after pre-treatment with gingipains (Olczak et al., 2001; Smalley et al., 2007, 2011). In fact, R-gingipains cleave hemoglobin to allow oxidation from ferrous to ferric iron thus facilitating release of heme and subsequent degradation of globin by K-gingipain. Free heme is then bound by HmuY. HmuY was proposed to be a hemophore-like protein because it was found either attached to the outer membrane or release in the supernatant. This release is dependent on proteolytic cleavage by gingipains (Wojtowicz et al., 2009). Once heme is bound to HmuY, it is transferred to HmuR for uptake. As with other outer membrane receptors, HmuR has two conserved His residues and the NPDL motif for heme binding and utilization (Liu et al., 2006). The *hmuY* and *hmuR* genes are regulated by the transcriptional activator PG1237 and are part of a larger locus (*hmuYRSTUV*) (Wu et al., 2009). The *hmuSTUV* genes may be responsible for heme transport to the cytoplasm. HmuS has sequence similarity to the cobN/Mg chelatase, HmuT and HmuU are similar to permeases and HmuW is annotated as an ATP-binding protein involved in hemin import (Lewis et al., 2006). Further studies are required to investigate these roles.

#### Gram-positive bacteria

The Isd (iron regulated surface determinant) system found in *Staphylococcus aureus* is one of the best-characterized mechanisms of iron acquisition from heme in Gram-positive bacteria. As illustrated in Figure 2, the Fur-regulated Isd machinery is composed of four cell wall-anchored proteins (IsdABCH), two cell wall sortases (SrtA and SrtB), a membrane transporter (IsdDEF) and two cytoplasmic heme oxygenases (IsdG and IsdI) (Mazmanian et al., 2003). Cell surface exposed IsdA binds heme, IsdB binds hemoglobin and heme, and IsdH binds heme, hemoglobin, haptoglobin and the complex of hemoglobin-haptoglobin (Dryla et al., 2003, 2007). Once heme is extracted by IsdH or IsdB, it is transferred unidirectionally to either IsdA or IsdC. Transfer can also occur from IsdA to IsdC, and bidirectionally between IsdH and IsdB. As well, IsdC transfers heme unidirectionally to the lipoprotein IsdE (Liu et al., 2008; Muryoi et al., 2008; Zhu et al., 2008). The IsdABCH proteins in *S. aureus* have been structurally characterized and found to all possess one or more NEAT domains. The NEAT domain is a poorly conserved 120 amino acid region that is encoded in variable numbers in genes located in the vicinity of putative siderophore transporter genes; NEAT therefore stands for near transporter (Andrade et al., 2002). NEAT domains can bind heme, hemoglobin, or hemoglobin-haptoglobin. As an example, IsdH possesses three NEAT domains (N1, N2, and N3) and it has been demonstrated that N1 and N2 bind hemoglobin and hemoglobin-haptoglobin, whereas N3 binds heme (Pilpa et al., 2009). It is thought that the transfer of heme across the cell wall of *S. aureus* occurs by protein-protein interactions that shuttle heme from one NEAT domain to another until the membrane is reached (Wandersman and Delepelaire, 2012). The heme molecule is believed to be transported across the inner membrane via the action of the ABC transporter IsdDEF. However, it has been shown that an *isdDEF* mutant does not significantly reduce heme use, suggesting that another ABC transporter might be present in *S. aureus* (Mazmanian et al., 2003). Nonetheless, iron is then released in the cytoplasm by degradation via the action of IsdG and IsdI, which have similarity to monooxygenases (Wu et al., 2005). Virulence assays revealed that an *isdB* mutant, but not an *isdH* mutant, showed reduced virulence in a murine abscess model of disease (Torres et al., 2006).

The Isd system has also been identified in several Gram-positive bacteria including *Streptococcus pyogenes* and *Bacillus anthracis* (Maresso et al., 2006; Nygaard et al., 2006). In the latter species, the Isd system is composed of three genes (*isdX1*, *isdX2*, and *isdC*) that encode proteins with one or more NEAT domains. It was shown that IsdX1 and IsdX2 are secreted proteins that extract heme from hemoglobin and deliver it to cell wall-bound IsdC (Fabian et al., 2009). The IsdX1 and IsdX2 proteins do not possess a cell-wall anchoring motif, and they are therefore thought to be secreted hemophores (Maresso et al., 2008). So far, it is unclear how heme is transported into the cell for *B. anthracis*. A new hemophore, Hal, has also been discovered recently in this bacterium (Balderas et al., 2012). Hal contains one NEAT domain that binds heme, the protein has several leucine-rich repeats and is proposed to be covalently coupled by a sortase to the cell wall via its C-terminal Gram-positive bacterium anchor (GPA). Deletion of *hal* resulted in a growth defect on heme or hemoglobin as the sole iron source (Balderas et al., 2012). Recently, another iron regulated leucine-rich surface protein (IlsA) was identified in *Bacillus cereus*. This protein has a conserved NEAT domain and directly binds heme. Inactivation of *ilsA* decreases the ability of the bacterium to grow in the presence of hemoglobin, heme, and ferritin, indicating a role in iron acquisition for IlsA. Moreover, the *ilsA* mutant showed a reduction in growth and virulence in an insect model, suggesting an important role for iron acquisition in disease caused by *B. cereus* (Daou et al., 2009).

A similar heme/hemoglobin uptake system (Shp-Shr-HtsABC) was found in *Streptococcus pyogenes*, where HtsABC encodes an ABC transporter, Shp binds heme on the cell surface and Shr binds hemoglobin and the hemoglobin/haptoglobin complex (Lei et al., 2002, 2003; Bates et al., 2003). Furthermore, the direct transfer of heme from hemoglobin by Shr to Shp has been demonstrated (Lu et al., 2012), and further characterization of Shp revealed two NEAT domains, a series of leucine-rich repeats and the absence of a cell wall-anchoring motif. It was also demonstrated that Shr spans the cell wall and is exposed to the extracellular environment, reminiscent of the Hal protein of *B. anthracis* (Fisher et al., 2008; Ouattara et al., 2010).

#### Mycobacterium tuberculosis

*M. tuberculosis* is known to acquire iron from transferrin and lactoferrin through secretion of the siderophores mycobactin and exochelins (Gobin and Horwitz, 1996). However, a pathway of heme utilization involving a secreted hemophore (Rv0203) and two trans-membrane proteins, MmpL11 and MmpL13, has been discovered recently. Mutation of either *rv0203* or *mmpL11* significantly reduces growth on heme or hemoglobin as a sole iron source, while mutation of *mmlp13* was unsuccessful and the gene may be essential (Tullius et al., 2011). It also has been shown that Rv0203 binds heme with a similar affinity constant to the heme binding proteins PhuS and HmuT from *P. aeruginosa* and *Y. pestis*, respectively (Owens et al., 2012). Upon binding, Rv0203 rapidly transfers heme to either of the inner membrane transporters MmpL11 and MmpL13 (Owens et al., 2013).

### Heme uptake by fungi

Much less is known about heme use by pathogenic fungi compared with bacterial pathogens. The ability to utilize heme and hemoglobin as an iron source by *C. albicans* was first described in 1992 (Moors et al., 1992). It was initially demonstrated that *C. albicans* binds erythrocytes via complement-receptor-like molecules (Moors et al., 1992). Subsequently, it was found that *C. albicans* possesses a hemolytic factor described as a secreted mannoprotein, although further characterization is needed for this factor (Watanabe et al., 1999). Nevertheless, the uptake of hemoglobin is mediated by specific receptors exposed on the surface of *C. albicans*, as illustrated in Figure 2. The first two heme/hemoglobin receptors to be identified were Rbt5 and Rbt51. Both of these are extracellular, glycosylphophatidylinositol (GPI)-anchored proteins and they harbor a conserved CFEM domain that may be involved in heme binding (Weissman and Kornitzer, 2004). CFEM domains are composed of eight cysteine residues of conserved spacing and they are found in a number of fungal membrane proteins (Kulkarni et al., 2003). Three other members of the hemoglobin-receptor family (Csa1, Csa2, and Pga7) have been identified based on the presence of the CFEM domain (Almeida et al., 2009). Rbt51 is sufficient by itself to confer the ability to use hemoglobin on *S. cerevisiae*, while a mutant of *RBT5* also showed a strong reduction of heme and hemoglobin use by *C. albicans* (Weissman and Kornitzer, 2004). Furthermore, Rbt5 facilitates the rapid endocytosis of hemoglobin into vacuoles in *C. albicans* cells. This endocytic process requires Myo5, a type I myosin that may be involved in endocytic vesicle scission, CaSla2, which is an actin-binding protein also required for endocytosis, an active vacuolar ATPase, and a member of the HOPS complex (CaVps41) (Weissman et al., 2008). Components of the ESCRT (endosomal sorting complex required for transport) system are also involved in the utilization of hemoglobin. ESCRT complex proteins are generally involved in transporting membrane proteins to the multivesicular body compartment and from there to the vacuole, where proteins are degraded (Hurley and Emr, 2006). Therefore, it was interesting that the ESCRT components are involved in heme/hemoglobin utilization, and that individual mutants of *C. albicans* (i.e., *vps2, vps23, vps24, vps38, vps36* and *snf7*) show a growth delay in the presence of hemoglobin (Weissman et al., 2008). It is not clear how heme and hemoglobin are processed upon Rbt5 binding and endocytosis, but it has been proposed that acidification of the vacuole might be sufficient to extract heme from hemoglobin. Heme degradation by the heme oxygenase CaHmx1 may occur in the vacuole or in the cytosol via transport of the heme molecule by a vacuolar transporter (Pendrak et al., 2004; Weissman et al., 2008). Importantly, CaHmx1 is required for full virulence in a mouse model of disseminated candidiasis (Navarathna and Roberts, 2010).

The pathogenic yeast *C. neoformans* is also able to grow on hemoglobin and heme as sole iron sources (Jung and Kronstad, 2008). *C. neoformans* secretes a 43 KDa serine proteinase that degrades hemoglobin and other substrates, although further characterization of this proteinase is needed (Yoo Ji et al., 2004). Information is starting to accumulate about heme use by *C. neoformans*. For example, an *Agrobacterium*-mediated T-DNA insertion screen for mutants with reduced growth on heme identified the ESCRT-I protein Vps23 as being important for iron acquisition from heme. Deletion of *vps23* resulted in growth defect on heme presumably due to a defect in endocytosis and proper sorting of the heme cargo (Hu et al., 2013). Recently, the first candidate hemophore in fungi was described in *C. neoformans*. This mannoprotein, Cig1, was shown to support iron acquisition from heme and to make a contribution to virulence in a mouse model of cryptococcal disease (Cadieux et al., 2013). However, the contribution of Cig1 to virulence was only evident in a mutant that also lacked a reductive, high affinity uptake system (described further below).

It is likely that other pathogenic fungi are able to use heme and hemoproteins. For example, the dimorphic pathogen *Histoplasma capsulatum* uses heme as a sole source of iron via a putative cell-surface receptor, although further studies are needed to elucidate the mechanism of heme uptake (Foster, 2002). It is also known that some important fungal pathogens, such as *A. fumigatus*, lack the ability to use heme as an iron source (Eisendle et al., 2003; Schrettl et al., 2004; Haas, 2012).

## Iron acquisition from transferrin, lactoferrin and ferritin

### Direct acquisition of iron from transferrin and lactoferrin in bacteria

**Figure 3:**
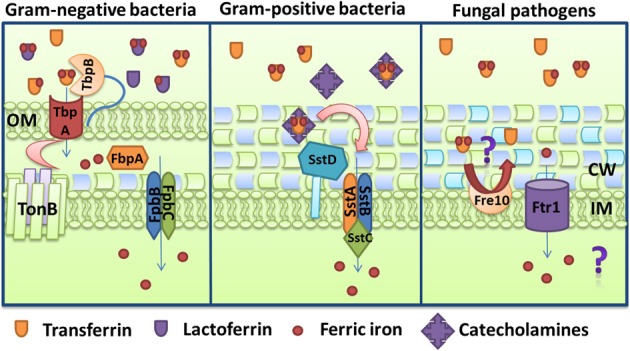
**Iron acquisition from transferrin and lactoferrin.** Mechanisms are shown for the Gram-negative bacterium *Neisseria gonorrhoeae* through the TbpAB-FbpABC transporter, and for the Gram-positive bacterium *Staphylococcus aureus* via the SstABCD transporter and catecholamines. For comparison, the uptake of iron that is potentially released from transferrin by the activity of the reductase Fre10 and the permease Ftr1 is also shown for the pathogenic fungus *Candida albicans*. The ferroxidase that functions with Ftr1 is not depicted. The descriptions of the specific proteins are given in the text.

Several bacterial pathogens can utilize non-heme, iron-containing proteins like transferrin, lactoferrin, and ferritins as sources of iron. As illustrated in Figure 3, the Gram-negative bacteria *N. meningitidis* and *N. gonorrhoeae* possess the receptors TbpAB and LbpAB that mediate the uptake of ferric iron from transferrin and lactoferrin, respectively (Cornelissen et al., 1992; Biswas and Sparling, 1995). The TbpAB system consists of two transferrin-binding proteins expressed from a biscistronic operon regulated by Fur and encoding the TonB-dependent protein TbpA and the lipoprotein TbpB that acts as a co-receptor (Ronpirin et al., 2001). TbpA binds apo and holo-transferrin with similar affinities, whereas TbpB only binds preferentially to iron-containing transferrin (Cornelissen and Sparling, 1996; Boulton et al., 1998). TbpA is able to extract iron from transferrin in the absence of its co-receptor, but the process is considerably more efficient in the presence of TbpB. In fact, it has been estimated that TbpB helps to internalize about half of the iron obtained from transferrin and also participates in the dissociation of apo-transferrin from the cell surface (Anderson et al., 1994; Derocco et al., 2009). The affinities for transferrin are distinct for TbpA and TbpB, and for the combined receptor (TbpAB), which suggests that formation of the dual receptor results in unique characteristics in the interaction with transferrin (Cornelissen and Sparling, 1996). Upon transferrin binding, TbpB forms a transient triple complex with TbpA. TbpA catalyzes a conformational change that leads to iron release and dissociation of apo-transferrin with the help of the TonB complex. TbpA is a TBDT protein and the conformational change moves the cork domain allowing the formation of a transient docking site for iron inside the β-barrel and transfer to the periplasmic ferric binding protein FbpA (Noinaj et al., 2012a,b). FbpA then initiates transport into the cytosol (Siburt et al., 2009).

FbpA is also known as the bacterial transferrin due to its similarities in structure and function to human transferrin (Parker Siburt et al., 2012). The *fbpABC* operon encodes an ABC transport system, where FbpB is a permease and FbpC is a nucleotide-binding protein that provides energy to transport iron across the cytoplasmic membrane (Adhikari et al., 1996; Strange et al., 2011). The FbpABC system is also involved in transport of iron from lactoferrin but is not required for the acquisition of iron from heme and hemoglobin (Khun et al., 1998). The FbpABC transporter is also required for the transport of xenosiderophores (i.e., siderophores such as enterobactin and salmochelin S2 from other microbes) in a TonB-independent fashion (see below) (Strange et al., 2011). Virulence was assessed in a murine model of *N. meningitidis* bacteremia, and both a *tbpA tbpB* mutant and a *tbpA* mutant are avirulent in mice suggesting a role for iron acquisition through transferrin in disease (Renauld-Mongenie et al., 2004). A *tbpB* mutant was as virulent as the wild-type strain. Importantly, a transferrin receptor mutant (Δ*tbpA* Δ*tbpB*) for *N. gonorrhoeae* was unable to initiate urethritis in human volunteers, demonstrating that a bacterial iron acquisition system is an essential virulence factor for human infection (Cornelissen et al., 1998). This bipartite receptor mechanism of iron acquisition from transferrin and lactoferrin is reminiscent of the heme bipartite receptor HupAB in *Neisseria* spp. and the hemophore Has system in *S. marcescens*. In addition, the use of an inner membrane ABC transporter is a recurrent mechanism shared by many pathogenic bacteria for iron transport.

The lactoferrin uptake system LbpAB in the *Neisseria* species is very similar to TbpAB in that LbpA is a TonB-dependent outer membrane protein and LbpB is a lipoprotein that serves as a co-receptor for LbpA (Biswas and Sparling, 1995). In contrast to the situation with TbpB and transferrin, LbpB is not required for uptake of iron from lactoferrin (Biswas et al., 1999). The specific mechanism of iron extraction from lactoferrin remains to be elucidated. Lactoferrin receptors are only found in about 50% of clinical isolates, whereas all isolates of *N. gonorrhoeae* express receptors that bind human transferrin. However, *in vivo* experiments demonstrated that the expression of the lactoferrin receptor in the absence of the transferrin receptor is sufficient for establishment of infection. Furthermore, in a mixed infection of male volunteers, expression of both lactoferrin and transferrin receptors gave a competitive advantage over a strain expressing only the transferrin receptor, thereby further indicating a role in virulence for iron acquisition from lactoferrin (Anderson et al., 2003).

### Involvement of catecholamines in iron acquisition from transferrin and lactoferrin

The availability of iron from transferrin and lactoferrin for bacterial use is also influenced by catecholamine stress hormones (epinephrine, norepinephrine and dopamine) and inotropes (isoprenaline and dobutamine) (Freestone et al., 2000, 2002; Neal et al., 2001; O'Donnell et al., 2006). Catecholamine stress hormones are able to bind transferrin and lactoferrin, to form direct complexes with ferric iron, and to reduce ferric to ferrous iron with subsequent liberation from transferrin (Sandrini et al., 2010). Free iron can then be used for bacterial growth via other specific iron uptake systems. This ability of stress hormones to mediate bacterial iron acquisition from transferrin and lactoferrin has been proposed to function in biofilm formation in intravenous lines by the Gram-positive bacterium *S. epidermidis* (Lyte et al., 2003). It may also play a role in the development of intra-abdominal sepsis by *E. coli* (Freestone et al., 2002) and be a contributing factor in biofilm formation on endotracheal tubing during ventilator-associated pneumonia caused by *P. aeruginosa* (Freestone et al., 2012).

For bacterial pathogens, iron acquisition involving catecholamines is mediated by siderophores or by mechanisms that are partially or completely independent of siderophore function. For example, enterohemorrhagic *E. coli* O157:H7 and *Salmonella enterica* can grow on transferrin in the presence of norepinephrine. The growth of both species in the presence of transferrin and norepinephrine also requires the synthesis, transport, and degradation of the siderophore enterobactin, suggesting that once iron is release from transferrin by a catecholamine, it is transported inside the bacteria by enterobactin. (Freestone et al., 2003; Methner et al., 2008). The transport of iron by enterobactin is discussed in more detail below. *Bordetella bronchiseptica* also uses catecholamines (norepinephrine, epinephrine, and dopamine) to obtain iron from both transferrin and lactoferrin (Anderson and Armstrong, 2008; Armstrong et al., 2012). The efficiency of iron acquisition from transferrin in the presence of catecholamine is increased by addition of enterobactin, but the siderophore is not essential, since norepinephrine alone can stimulate growth in presence of transferrin. This growth stimulation is dependent on TonB because a mutation in *tonB* abolishes growth in presence of transferrin and NE (Anderson and Armstrong, 2008). A genetic screen identified three TonB-dependent outer membrane receptors (BfrA, BfrD, and BfrE) for catecholamines that are required for growth in the presence of catecholamines and transferrin. These receptors can also mediate the uptake of enterobactin and 2,3-dihydroxybenzoic acid. The characterization of catecholamine-mediated iron uptake for *B. bronchiseptica* revealed a siderophore-independent pathway. However, its features imply that siderophores may act to shuttle iron between transferrin and outer membrane receptors (Armstrong et al., 2012).

Growth stimulation by norepinephrine in the presence of transferrin has been also shown to be independent of siderophore production for *E. coli* and *Bacillus subtilis* (Miethke and Skerra, 2010). Mutants with defects in siderophore biosynthesis in both bacteria are still able to grow in the presence of norepinephrine and transferrin, indicating that iron-complexed norepinephrine can directly serve as an iron source. However, the FeuABC uptake system for bacillibactin was also identified in *B. subtilis* to be involved in the use of iron-complexed norepinephrine, since deletion of this locus abolished growth stimulation by NE and transferrin (Miethke et al., 2006). Furthermore, this iron acquisition could be abolished by the addition of siderocalin, the host innate immune protein that binds enterobactin and inhibits its use by the bacteria (Miethke and Skerra, 2010). A similar system may operate in other Gram-positive bacteria because a siderophore-deficient strain of *S. aureus* can grow in human serum in the presence of catecholamines (epinephrine, norepinephrine, and dopamine). In this case, iron uptake via catecholamine sequestration is mediated by the transporter SstABCD, as shown in Figure 3. Based on sequence similarities, the *sst* genes encode two putative cytoplasmic membrane proteins (SstA and SstB), an ATPase (SstC), and a membrane-bound lipoprotein (SstD) (Morrissey et al., 2000). Moreover, *S. aureus* can use its endogenous siderophores, staphyloferrin A and staphyloferrin B, to access the transferrin iron pool (Beasley et al., 2011). The collective activities of the siderophore transporters (Hts and Sir) and the Sst transport system are required for full virulence of *S. aureus* in intravenously challenged mice. However, *sst* inactivation was sufficient to significantly decrease colonization of the mouse heart (Beasley et al., 2011).

### Fungal acquisition of iron from transferrin and lactoferrin

Transferrin and lactoferrin are known to have an inhibitory effect on the growth of the pathogenic fungi *A. fumigatus*, *C. albicans* and *C. neoformans* (Sridhar et al., 2000; Ahluwalia et al., 2001; Lahoz et al., 2008; Almeida et al., 2009; Kornitzer, 2009; Okazaki et al., 2009). The mechanism of inhibition is probably due to iron sequestration by partially iron-loaded protein because additional studies have shown that these fungi can acquire iron from fully iron-loaded transferrin under specific conditions. For example, iron-loaded transferrin, but not apo-transferrin, restores growth to iron-starved cells of *C. albicans* (Knight et al., 2005). In this fungus, the use of transferrin iron is dependent on fungal contact with the transferrin and on a reductive, high affinity uptake system that includes the iron permease Ftr1 and a reductase Fre10 (Figure 3). Importantly, Ftr1 is required for virulence thus suggesting iron acquisition from transferrin during infection (Ramanan and Wang, 2000). Siderophore and heme uptake systems did not play a role in iron acquisition from transferrin by *C. albicans*. In contrast, *A. fumigatus* uses secreted siderophores to obtain iron from transferrin and this may be important during disease (Hissen et al., 2004; Hissen and Moore, 2005; Haas, 2012). The situation for *C. neoformans* resembles that of *C. albicans* where an iron permease, Cft1, of the reductive, high affinity system is required for iron use from transferrin and for full virulence (Jung et al., 2008).

### Iron acquisition from ferritins

Ferritins represent a potentially rich source of iron for bacteria and fungi. For example, *N. meningitides* is able to use iron from ferritin after a rapid redistribution and degradation of cytosolic ferritin in infected epithelial cells (Larson et al., 2004). Ferritin is in fact aggregated and recruited by intracellular meningococci and degradation of ferritin provides an excellent source of iron (Larson et al., 2004). For the fungi, ferritin use as a sole iron source has been best characterized for *C. albicans*. This pathogen uses the adhesin Als3 as a ferritin receptor, as demonstrated by the findings that deletion of *als3* blocks ferritin binding and that heterologous expression of Als3 in *S. cerevisiae* confers the ability to bind ferritin (Almeida et al., 2008).

## Ferric iron acquisition by siderophores

Many bacteria and fungi (and perhaps mammals) produce siderophores (low molecular weight, high affinity ferric chelators) to acquire and transport iron, as detailed in several reviews (Andrews et al., 2003; Miethke and Marahiel, 2007; Winkelmann, 2007; Haas et al., 2008). The first three siderophores were isolated and identified from bacteria (mycobactin and coprogen) and fungi (ferrichrome). Snow and collaborators first reported in 1949 that supplementation with purified mycobactin enhanced the growth of *Mycobacterium johnei* (also known as *M. paratuberculosis*) (Francis et al., 1949). Mycobactin was considered to be a growth factor, although a high affinity for ferric chloride was also noted (Francis et al., 1953; Snow, 1954). Early experiments identified other growth factors with apparently dissimilar structures but strong chelating activity for ferric iron, including the Terregens Factor (later identified as arthrobactin), coprogen, and ferrichrome (Hesseltine et al., 1952; Lochead et al., 1952; Neilands, 1957). Garibaldi and Neilands reported the key finding that the production of ferrichrome A was enhanced when the fungus *Ustilago sphaerogena* was grown in iron-depleted medium, and that several other microbes (e.g., the bacteria *B. subtilis* and *Bacillus megaterium*, and the fungus *Aspergillus niger*) produced iron-binding compounds under similar conditions (Garibaldi and Neilands, 1956). This work led to the suggestion that the growth factors might be involved in a system for sequestering and transferring iron that is induced during iron deficiency. This key observation led to a refined view of the function of siderophores and their biological significance. In fact, siderophores enhance growth by coordinating ferric iron for uptake by microorganisms using facilitative transport machinery.

Numerous reviews have appeared describing the types of siderophores produced by microbes (Crosa and Walsh, 2002; Winkelmann, 2002, 2007; Andrews et al., 2003; Miethke and Marahiel, 2007; Haas et al., 2008). Therefore, we will focus on selected principles and examples for bacterial and fungal pathogens to illustrate general properties. Importantly, in addition to a role in iron acquisition in the context of infection, some siderophores are secreted by microorganisms to deprive competing organisms of iron (Emery, 1982). Conversely, many microorganisms have evolved the transport machinery to use heterologous siderophores produced by other microbes (xenosiderophores) (Winkelmann, 2007). This is the case for opportunistic pathogen *P. aeruginosa* which produces two different siderophores, pyoverdine and pyochelin (Cox, 1980; Cox and Adams, 1985), but can utilize a variety of heterologous siderophores from other bacteria and fungi, including ferrioxamine B, ferrichrome and enterobactin (Poole et al., 1990; Cuiv et al., 2007). A focus on the use of xenosiderophores is also the case for the fungal pathogens *C. albicans* and *C. neoformans*, as described below. Of course, many pathogenic microorganisms produce siderophores that are directly implicated in their virulence (Miethke and Marahiel, 2007; Garenaux et al., 2011). In this case, siderophores of bacterial and fungal pathogens can directly remove iron from host proteins such as transferrin to support proliferation in vertebrates (Konopka et al., 1982; Brock et al., 1983).

### Enterobactin, the archetypical siderophore

The archetypical bacterial siderophore is the catecholate enterobactin, also known as enterochelin. This siderophore was identified simultaneously by O'Brien and Gibson (1970), who isolated enterochelin from *E. coli*, and Pollack and Neilands (1970), who characterized enterobactin from *S. enterica* Typhimurium. Enterobactin has been extensively studied over the past 40 years and it is the siderophore with the strongest known affinity for ferric iron (*K*_d_ of 10^−52^M) (Harris et al., 1979). Enterobactin participates in the retrieval of iron from transferrin, as discussed earlier, and the siderophore is produced by *E. coli*, *Salmonella spp*., *Klebsiella spp*, and by some strains of *Shigella* (Wyckoff et al., 2009). Enterobactin can, however, be sequestered by the host innate immune protein siderocalin (also known as lipocalin 2) as a defense mechanism to prevent bacteria from accessing iron (Goetz et al., 2002; Flo et al., 2004). In response, the pathogenic enterobacteria don't rely solely on enterobactin to gain access to iron within the host and they possess multiple siderophore systems. In particular, enterobactin can be modified into salmochelins by the addition of up to three glucose molecules on its catechol moieties (Hantke et al., 2003; Bister et al., 2004). This glycosylation blocks binding by siderocalin without altering iron binding by the siderophore (Fischbach et al., 2006). Hence, the production of salmochelins contributes to virulence of pathogenic *E. coli*, *S*. Typhimurium, and *K. pneumoniae* (Caza et al., 2008, 2011; Crouch et al., 2008; Bachman et al., 2012). Two other types of siderophores can be produced by these bacteria, aerobactin and yersiniabactin, and these can also escape siderocalin sequestration and contribute to the virulence of pathogenic *E. coli* and *K. pneumoniae* (Dozois et al., 2003; Fischbach et al., 2006; Bachman et al., 2011; Correnti and Strong, 2012).

A common observation is that pathogens often deploy multiple iron acquisition systems or siderophores to support proliferation in the host (Dozois et al., 2003; Garenaux et al., 2011; Kronstad et al., 2013). In particular, redundancy in siderophore iron acquisition systems can mask the contribution of each individual system to virulence. A good example comes from the production of pyochelin and pyoverdine by *P. aeruginosa*. In an intramuscular infection model with immuno-compromised mice, only the strain mutated for the production of both pyochelin and pyoverdine showed attenuation of virulence. However, in an intranasal murine model of infection, only pyoverdine is required for pathogenesis, although loss of both molecules more severely attenuated virulence (Takase et al., 2000).

### Siderophore transport in gram-negative bacteria

Typically, the internalization of siderophores in bacteria is facilitated by ABC type transporters. Although in some cases, inner membrane permeases driven by energy proton motrive force can also translocate iron-loaded siderophores. The iron-loaded siderophore is first recognized and internalized by specific cell-surface receptors, which are all members of the TBDT family and are usually regulated by Fur. The ferri-siderophore is then processed through the different membranes and the cell wall by chaperone proteins and facilitators. Once the molecule reaches the intracellular space, the iron atom can be released by physical degradation of the siderophore or by a redox-mediated process, the affinity of siderophores for ferrous iron being much less than that for ferric iron. In some cases, such as with pyoverdine uptake by *P. aeruginosa*, iron can be released in the periplasmic space with subsequent transport of siderophore-free iron into the cytoplasm and recycling of the empty siderophore to the extracellular medium (Faraldo-Gomez and Sansom, 2003; Wandersman and Delepelaire, 2004; Schalk and Guillon, 2013).

**Figure 4:**
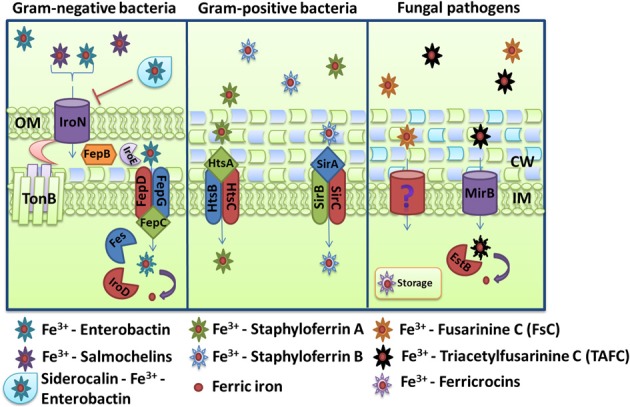
**Schemes for ferric iron uptake via siderophores.** The receptor IroN, the ABC-transporter FepBCDG and the esterases Fes, IroD, and IroE mediate the uptake of iron-loaded enterobactin and salmochelins in the Gram-negative bacterium *Escherichia coli*. For *Staphylococcus aureus*, the ABC transporters HstABC and SirABC perform the uptake of the siderophores staphyloferrin A and staphyloferrin B, respectively. The fungus *Aspergillus fumigatus* secretes the siderophores FsC and TAFC, and the major facilitator superfamily protein MirB is known to transport TAFC for subsequent degradation by the EstB. The descriptions of the specific proteins are given in the text.

For Gram-negative bacteria, iron-loaded siderophores need to pass two membranes and a peptidoglycan cell wall to reach the intracellular space (Figure 4). Recognition and internalization requires specific receptors on the cell surface and examples include FepA, IroN, and PfeA from *E. coli*, *S. enterica* and *P. aeruginosa*, respectively (Lundrigan and Kadner, 1986; Dean and Poole, 1993; Hantke et al., 2003). A single transport system can also internalize different siderophores. For example, the internalization of the siderophore aerobactin in *E. coli* is supported by the receptor IutA and the ABC transporter FhuBCD (De Lorenzo et al., 1986; Wooldridge et al., 1992). This transporter also mediates the uptake of ferrichrome, coprogen and rhodotorulic acid with the help of the specific receptors FhuA, FhuE, and Fiu (Fecker and Braun, 1983; Hantke, 1983). This illustrates the versatility of receptor-substrate recognition and also the piracy for iron acquisition that exists among competitive pathogens. The detailed processes of siderophore internalization are illustrated in Figure 4 for the well-characterized mechanism of the fur-regulated catecholate siderophores system, enterobactin, and salmochelins. Iron-loaded catecholate siderophores are translocated upon recognition by the outer membrane receptor FepA (for enterobactin only) or IroN coupled to the energy transducing TonB-ExbD-ExbB complex (Pierce et al., 1983). After internalization, ferri-siderophore moves through the inner membrane. This passage requires proteins located in the periplasmic space and an inner membrane transporter. Cyclic molecules can be linearized in the periplasm by the esterase IroE (Lin et al., 2005; Zhu et al., 2005). The periplasmic protein FepB and the ABC transporter FepCEG translocate iron-loaded siderophores into the bacterial cytoplasm (Shea and McIntosh, 1991; Sprencel et al., 2000; Crouch et al., 2008). Once in the cytoplasm, the release of iron requires degradation of the molecule. The esterases Fes and IroD cleave iron-loaded enterobactin and salmochelins at ester bonds creating monomers, dimers, and trimers of DHBS and their glycosylated versions (Langman et al., 1972; Lin et al., 2005). These molecules can then be resecreted outside the bacteria, via their specific efflux pump EntS and IroC and reutilized as siderophores (Caza et al., 2011). This recycling characteristic of siderophore molecules is similar to the recycling of transferrin receptors and hemophores.

### Siderophore transport in gram-positive bacteria

Siderophore transport in gram-positive bacteria is similar to the process in Gram-negative bacteria in that ABC transporters mediate translocation into the cytoplasm. The system in *S. aureus* provides a good illustration of the process. This bacterium produces for two siderophores, staphyloferrin A and staphyloferrin B, which are transported into the cytoplasm through the ABC transporters HtsABC and SirABC, respectively (Figure 4) (Meiwes et al., 1990; Beasley et al., 2009). HtsA and SirA are receptors exposed on cell surface while HtsBC and SirBC are components in the membrane responsible for the transport into the cell (Beasley et al., 2011). HtsBC also participates in the uptake of heme, suggesting a dual role for the HtsABC transporter (Skaar et al., 2004). The *sfa* and *sbn* loci encode the enzymes for staphyloferrin A and staphyloferrin B biosynthesis, respectively, and are regulated negatively by Fur and iron (Beasley et al., 2009, 2011). As discussed earlier, these siderophores are able to acquire iron from transferrin and lactoferrin with the help of catecholamine, although they are also able to mediate the uptake of ferric iron directly. In addition, *S. aureus* can utilize exogenous hydroxamate siderophores like aerobactin, ferrichrome, ferrioxamine B and coprogen through the Fhu (FhuCBG, FhuD1and FhuD2) uptake system (Sebulsky et al., 2000; Sebulsky and Heinrichs, 2001). FhuB and FhuG are membrane components and FhuC is the ATP-binding protein. FhuD1 and FhuD2 are lipoproteins thought to function as binding proteins for hydroxamate siderophores and staphylobactin (Sebulsky et al., 2003). Assays with a Δ *fhuCBG* mutant revealed a significant contribution to virulence in a murine kidney abscess model (Speziali et al., 2006).

*Listeria monocytogenes* provides a useful additional example because this facultative intracellular pathogen uses several iron uptake systems. It can acquire iron from host proteins such as transferrin, lactoferrin, ferritin, and hemoglobin, but it does not secrete any siderophores. Rather it can use several hydroxamate (ferrichrome, ferrichrome A and ferrioxamine B) and catecholate (enterobactin and corynebactin) siderophores from other organisms and it can use additional iron-binding compounds, including catecholamines (Simon et al., 1995; Jin et al., 2006). As in *S. aureus*, the ABC transporter FhuCDBG system in *L. monocytogenes* is responsible for uptake of the hydroxamate siderophore ferrichrome and the HupDGC transporter mediates the uptake of iron from hemoglobin (Jin et al., 2006).

### Siderophore production and transport in pathogenic fungi

As a group, fungi produce a number of structurally different siderophores and, as mentioned, some of the earliest studies of siderophores involved ferrichrome and ferrichrome A (Burnham and Neilands, 1961; Zalkin et al., 1964). The ferrichrome siderophore family illustrates the potential for complexity because it consists of 20 structurally different hexapeptides where modifications can occur on a common ferrichrome backbone molecule. These modifications include the addition, for example, of a hydroxymethyl group, a methyl group, or a lateral side chain. These alterations can generate derivatives such as ferricrocin, ferrichrysin, asperchrome D1 and B1, ferrirubin, ferrirhodin, ferrichrome A and other molecules (Winkelmann, 2007). At the other end of the spectrum, there are also fungi that do not produce any known siderophores (as with *L*. *monocytogenes* discussed above), but readily make use of xenosiderophores through the deployment of specific transporters. In general, fungi use transporters of the major facilitator protein superfamily, rather than ABC transporters, for siderophore internalization (Haas et al., 2003, 2008).

The importance of siderophores in fungal virulence in humans is nicely illustrated by detailed studies with the airborne pathogen *A. fumigatus* (and parallel comparative studies with the related saprotrophic species *Aspergillus nidulans*) (Eisendle et al., 2003; Schrettl et al., 2004, 2007; Haas, 2012)*. A. fumigatus* produces the siderophores fusarinine C (FsC)/triacetylfusarinine C (TAFC) and ferricrocin, and much is known about the regulation, biosynthesis, uptake and role in virulence for these molecules (Hissen et al., 2004; Schrettl et al., 2004, 2007; Kragl et al., 2007; Wallner et al., 2009; Haas, 2012). FsC and TAFC are excreted in response to iron deprivation and they function in extracellular iron binding with subsequent uptake by siderophore iron transporters (SITs) (Figure 4). *A. fumigatus* is predicted to encode 10 SITs (Haas, 2012) and two from *A. nidulans* have been functionally characterized: MirA was found to transport enterobactin and MirB was shown to take up TAFC in both *A. nidulans* and *A. fumigatus* (Haas et al., 2003; Raymond-Bouchard et al., 2012). After internalization, the intracellular release of iron from TAFC is achieved by hydrolysis of the siderophore backbone by the esterase EstB (Kragl et al., 2007). Interestingly, *A. fumigatus* possesses ferricrocin intracellular siderophores, and their production is coordinated with the morphology of the fungus. That is, ferricrocin (FC) is produced during filamentous hyphal growth, while hydroxyferricrocin (HFC) is produced within the conidial spores that are the infectious particles (Schrettl et al., 2007; Wallner et al., 2009). The intracellular siderophores are believed to function in iron storage (Schrettl et al., 2007; Wallner et al., 2009). Both intracellular and extracellular siderophores contribute to the virulence of *A. fumigatus* because the deletion of key genes for production results in avirulence in a murine model of invasive pulmonary aspergillosis (Schrettl et al., 2007). A recent study demonstrated that topical treatment with the human tear lipocalin (TL, also known as Lcn1) or lactoferrin reduced *A*. *fumigatus* growth in the cornea of mice, suggesting that therapeutic inhibition of fungal iron acquisition can be used to treat infections (Leal et al., 2013). TL is a secretory protein that interferes with microbial growth by scavenging microbial siderophores. In contrast to siderocalin, TL binds to a broader array of siderophores, including fungal siderophores such as coprogen, TAFC and rhodotorulic acid (Fluckinger et al., 2004).

The pathogenic yeasts *C. albicans* and *C. neoformans* don't produce siderophores but can scavenge xenosiderophores from other microbes. This iron parasitism depends on specific siderophore transporters in the plasma membrane. For example, the transporter Sit1 (also designated Sit1p/Arn1p) from *C. albicans* mediates the uptake of ferrichrome-type siderophores including ferricrocin, ferrichrysin, ferrirubin, coprogen and TAFC (Heymann et al., 2002). A mutant lacking Sit1 had a reduced ability to damage cells in a reconstituted human epithelium model of infection (Heymann et al., 2002). In *C. neoformans*, the transporter Sit1 is required for the uptake of ferrioxamine B, but does not make a contribution to virulence in a mouse model of cryptococcosis (Tangen et al., 2007).

## Uptake of ferrous iron

Activities for the reduction of ferric iron and subsequent uptake of ferrous iron are present in bacteria and fungi. The ferrous form can exist in acidic environments and under anoxic conditions, and it can be generated by cell-associated or exported reductase activities. Ferrous iron ions are believed to diffuse freely through the outer membrane of Gram-negative bacteria, with subsequent transport through the inner membrane by the ABC transporter FeoABC. This system is conserved in many species, and it was first discovered in the non-pathogenic *E. coli* strain K-12 (Kammler et al., 1993). FeoB is the main transmembrane transporter that acts as a permease, while FeoC has been proposed to regulate FeoB. The role of FeoA is not well-understood, but it interacts with the highly conserved core region of FeoB (Lau et al., 2013). This system is under control of *fnr* and *fur* regulatory elements, where Fnr is an anaerobically-induced transcriptional activator and Fur inhibits transcription of *feo* genes in iron-replete conditions (Spiro and Guest, 1990; Kammler et al., 1993). The Feo system also contributes to intracellular replication for facultative intracellular pathogens like *Legionella pneumophila* (Robey and Cianciotto, 2002), *Shigella flexneri* (Runyen-Janecky et al., 2003) and *Francisella tularensis* (Thomas-Charles et al., 2013).

**Figure 5:**
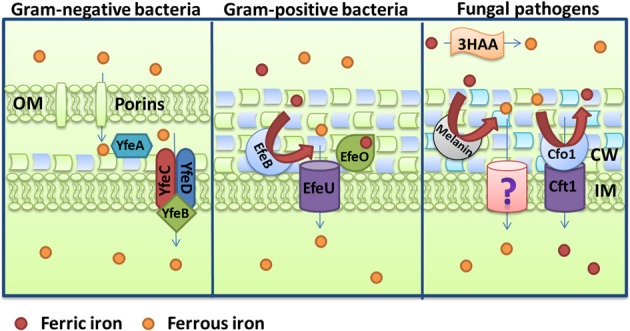
**Schemes for ferrous iron uptake.** The Gram-negative bacterium *Yersinia pestis* uses the YfeABCD proteins and the Gram-positive bacterium *Bacillus subtilis* uses the EfeUOB complex to accomplish ferrous iron uptake. A comparable process is shown for the fungal pathogen *Cryptococcus neoformans*. This pathogen used the Cfo1-Cft1 multicopper oxidase-iron permease complex, the cell wall pigment melanin, and the secreted reductant 3-hydroxyanthranilic acid to perform reduction and ferrous iron uptake. Note that ferrous iron is oxidized by Cfo1 prior to transport into the cell by Cft1. Physiological evidence for an additional low affinity transport system for ferrous iron has been presented for *C. neoformans* and this is indicated by a question mark (Jacobson et al., 1998). Additional details for each system are provided in the text.

Other ferrous iron transport systems are present in various bacterial species. For example, the SitABCD system exists in *S. enterica* and *E. coli*, and a similar system has been described in *Y. pestis* (YfeABCD) (Bearden and Perry, 1999). The Yfe system is a typical ABC transporter for ferrous iron and manganese. YfeA is a periplasmic binding protein, YfeC and YfeD are the two inner membrane permeases, and YfeB is the ATPase required for energy transduction transport. No outer membrane protein has been identified and transport is independent of TonB (Figure 5) (Bearden et al., 1998; Perry et al., 2003; Fetherston et al., 2010). The Fur-regulated EfeUOB system is found in enterohaemorrhagic *E. coli* O157:H7 and *E. coli* Nissle 1917, and is also thought to transport ferrous iron (Grosse et al., 2006; Cao et al., 2007). This system promotes growth under aerobic, low-pH and low-iron conditions in response to ferrous iron (Cao et al., 2007). Interestingly, the EfeUOB system is analogous to the reductive, high-affinity iron uptake system of *S. cerevisiae* (Ftr1-Fet3-Fre1). Frt1 is an iron permease (with homology to EfeU), Fet3 is a multicopper oxidase (MCO) that will oxidize ferrous iron to its ferric state during the translocation and Fre1 is one of seven reductases that reduce ferric iron (Kosman, 2003). The EfeUOB system also exists in the Gram-positive bacterium *B. subtilis* where it permits acquisition of both ferrous and ferric iron species depending on extracellular conditions (Miethke et al., 2013). The ferrous iron peroxidase EfeB and the ferric iron binding protein EfeO act in succession during ferrous oxidation and ferric iron delivery to the membrane permease EfeU. However, EfeB is dispensable for direct ferric uptake via EfeUO, but instead promotes growth under microaerobic conditions where ferrous iron is more abundant. EfeUOB thus has a dual mechanism for acquisition of iron (Figure 5) (Miethke et al., 2013). More recently, the FtrABCD system for ferrous iron transport has been characterized in *Bordetella pertussis* and *Bordetella bronchiseptica*, and in *Brucella abortus* (Brickman and Armstrong, 2012; Elhassanny et al., 2013). Importantly, a *B. abortus* mutant lacking FtrA is attenuated in murine macrophages and mice thereby indicating the importance of ferrous iron in the mammalian host.

As mentioned above for the yeast *S. cerevisiae*, fungi have a high affinity system consisting of reductases, an iron permease and a MCO to generate ferrous iron for uptake, and this is the case for *A. fumigatus*, *C. albicans*, and *C. neoformans*. The components of this system and its contribution to iron acquisition in a vertebrate host were first characterized for *C. albicans*. Two *C. albicans* reductases, Cfl1 and Cfl95, were identified that promote reduction of ferric iron upon heterologous expression in a *S. cerevisiae* reductase-deficient strain. A large number of additional reductases are predicted from the *C. albicans* genome sequence (Hammacott et al., 2000; Knight et al., 2002). As in *S. cerevisiae*, reduced iron is transported into the cell by a complex consisting of an MCO and a permease. Five MCO candidates are predicted for *C. albicans*, and *CaFET3*, *CaFET31* and *CaFET43*, can rescue the growth of a *fet3*Δ (MCO) mutant of *S. cerevisiae* in response to iron limited conditions (Ziegler et al., 2011; Cheng et al., 2013). Moreover, deletion of *CaFET33* and *CaFET34* decreased cellular iron content and iron acquisition during iron starvation, and *CaFET3* can compensate for the loss of *CaFET33* and *CaFET34* (Cheng et al., 2013). *C. albicans* has two iron permeases, *CaFTR1* and *CaFTR2*. The expression of *CaFRT1* is induced by iron starvation and this gene is required for iron acquisition from ferritin and transferrin (Ramanan and Wang, 2000; Almeida et al., 2009). A mutant that lacks the gene cannot cause damage to oral epithelial cells and, as mentioned earlier, is unable to cause systemic disease in a mouse model of candidiasis (Ramanan and Wang, 2000).

A screen of the *A. fumigatus* genome revealed 15 putative reductases, and FreB was shown to participate in adaptation to iron starvation and to function as a reductase (Blatzer et al., 2011). In this pathogen, the reduced ferrous iron is then re-oxidized by the MCO FetC and imported by the iron permease FtrA; FetC and FtrA are 52% and 55% identical to the *C. albicans* Fet3 and Frt1 proteins, respectively (Schrettl et al., 2004). However, in *A. fumigatus*, the reductive iron uptake system does not play a role in virulence, and the siderophore system appears to be much more important for proliferation in the host (Schrettl et al., 2007; Haas, 2012).

The situation in *C. neoformans* is similar to that of *C. albicans*. Reductase activities have been characterized and the MCO Cfo1 as well as the iron permease Cft1 are required for reduction of iron from transferrin (Jung et al., 2009). Interestingly, in addition to enzymatic reductase activity, two other reduction systems exist at the cell surface for *C. neoformans*, the secreted reductant 3-hydrozyanthranilic acid (3HAA) and melanin which is responsible for a black cell wall pigmentation in presence of L-DOPA (Figure 5) (Nyhus et al., 1997). Cfo1 and Cft1 are both required for full virulence of *C. neoformans* in an inhalation murine model of cryptococcosis, however, mutants lacking these enzymes still cause disease (Jung et al., 2008, 2009). Therefore, additional iron acquisition functions are needed during disease. One of these functions includes the mannoprotein Cig1 that was recently shown to participate in heme uptake, as described earlier (Cadieux et al., 2013).

## Conclusions and perspectives

Pathogenic bacteria and fungi have evolved a number of mechanisms to acquire iron from different sources in the mammalian host. Although many of these mechanisms share functional similarities, it is clear that far more is known about bacterial systems. This is particularly evident for mechanisms that mediate iron acquisition from heme and heme-containing proteins. Considered as a group, key components have been identified in several bacterial pathogens and these include hemolysins, hemophores, receptors, ABC transporters for internalization and heme oxygenase activities. In contrast, the components that perform analogous functions in fungal pathogens are just now being identified and characterized. While there is some information about hemolysins and receptors, a candidate hemophore has only recently been described in a fungal pathogen and the details of uptake via endocytosis require considerable more investigation.

For other iron-containing host proteins, such as transferrin, lactoferrin and ferritin, there are clear differences between bacterial and fungal pathogens, although again the lack of information for fungi precludes detailed comparisons. It is clear that some bacteria, particularly *Neisseria* species, have sophisticated mechanisms for using transferrin, lactoferrin, and ferritin iron. In addition, there is a fascinating body of information on the participation of catecholamines in bacterial iron acquisition. This aspect of iron acquisition remains to be explored in fungal pathogens. For fungi, transferrin and lactoferrin tend to inhibit growth by an iron sequestration mechanism, although some fungi can overcome this limitation by reductive iron uptake or siderophore elaboration. Reductive iron uptake systems show similarities between bacteria and fungi in the uptake of ferrous iron, and this mechanism is clearly important for virulence in some but not all fungi. In addition, the recent discovery of a ferritin receptor in *C. albicans* has generated considerable interest in fungal exploitation of this iron source.

Siderophore-mediated acquisition of iron is one area where fungi as a group (i.e., not just pathogens) have provided significant structural and mechanistic information in parallel with studies in bacterial pathogens. In particular, the detailed studies in *A. fumigatus* (and the related species *A. nidulans*) on siderophore biosynthesis rival the sophisticated and advanced state of analysis for bacteria. However, the critical area of siderophore transport needs considerable attention for fungal pathogens. This is because little information is available for any of the species and because some of the most important species, *C. albicans* and *C. neoformans*, rely on transporters to steal siderophores. Considerable attention is now directed at siderophore-based drug development where siderophore transporters might be exploited as Trojan horse delivery systems. Therefore, an understanding of fungal siderophore transporters might facilitate the application of these drugs to fungal diseases. It is evident, however, that pathogenic bacteria and fungi generally possess more than one mechanism for exploiting the potential iron sources in vertebrate hosts. This is clear from virulence studies that often reveal only partial attenuation upon loss of a single uptake mechanism. Therefore, therapeutic approaches that target iron acquisition must inactivate the most critical of these mechanisms and/or exploit them for the delivery of antibacterial and antifungal drugs.

### Conflict of interest statement

The authors declare that the research was conducted in the absence of any commercial or financial relationships that could be construed as a potential conflict of interest.
